# Fetomaternal Outcome in Women With Gestational Diabetes Mellitus With and Without Polycystic Ovary Syndrome: A Comparative Approach

**DOI:** 10.7759/cureus.84256

**Published:** 2025-05-16

**Authors:** Rashmi Solanke, Vinita Singh, Lavanya Simma, Aarti Kumari, Priya Kumari

**Affiliations:** 1 Obstetrics and Gynaecology, All India Institute of Medical Sciences Raipur, Raipur, IND; 2 Obstetrics and Gynaecology, Government Medical College Nagpur, Nagpur, IND

**Keywords:** gestational diabetes mellitus, hyperandrogenism, hyperinsulinemia, polycystic ovarian syndrome, rotterdam criteria

## Abstract

Background

Gestational diabetes mellitus (GDM) is an increasing health concern during pregnancy, associated with various maternal and fetal complications, such as pre-eclampsia, polyhydramnios, fetal macrosomia, birth trauma, neonatal metabolic complications, and prenatal death. Polycystic ovary syndrome (PCOS) is characterized by symptoms like amenorrhea, oligomenorrhea, hirsutism, obesity, infertility, anovulation, and acne. This paper compares the fetomaternal outcomes in patients with both PCOS and GDM and those with GDM alone.

Aim

The aim of this paper is to compare the fetomaternal outcomes in patients with both PCOS and GDM with those having GDM alone. By identifying differences in outcomes, the study could help develop guidelines for early interventions and precautionary measures for women with PCOS before conception. This approach could reduce pregnancy complications and mitigate long-term metabolic risks for both the mother and the child. Furthermore, the paper emphasizes the need to develop predictive algorithms based on risk factors to prevent adverse outcomes for both mothers and neonates.

Materials and methods

This prospective cohort study included 88 singleton pregnancies with a known diagnosis of GDM, including those with PCOS diagnosed according to the Rotterdam criteria. Participants were women aged 18 years and older, with a gestational age between 5 and 40 weeks. The participants were divided into two groups: GDM with PCOS (Group A) and GDM without PCOS (Group B). At the first visit, patients were initially screened using the DIPSI criteria, which involves a non-fasting 75g glucose challenge test. Patients with a two-hour plasma glucose level of 140 mg/dL or above were identified as positive. Subsequently, these patients underwent a standard oral glucose tolerance test for further evaluation and confirmation of glucose intolerance. Women were followed until delivery. Various parameters were studied, including Doppler changes, polyhydramnios/oligohydramnios, gestational age, mode of delivery, onset of labor (spontaneous/induced), fetal growth restriction or macrosomia, shoulder dystocia, and puerperal complications such as postpartum hemorrhage, lactation failure, and breast abscess. Statistical analysis was performed using IBM SPSS Statistics for Windows, Version 20 (Released 2011; IBM Corp., Armonk, New York, United States). Continuous variables were expressed as mean ± SD, and categorical variables were represented as percentages. A two-sided P-value of less than 0.05 was considered statistically significant.

Conclusion

The study concluded that the patients in the GDM with PCOS group did not have poorer fetomaternal outcomes compared to those with GDM alone. Therefore, while certain factors like maternal weight gain and family history of diabetes differed between the groups, the presence of PCOS alongside GDM did not result in worse outcomes for either the mother or the neonate in this cohort.

## Introduction

Gestational diabetes mellitus (GDM) is glucose intolerance with the onset or first recognition in pregnancy. It is detected in 10-15% of pregnancies [1]. Women with a history of GDM are at increased risk of developing type 2 diabetes later in life. According to American College of Obstetrics and Gynecology (ACOG), it is any degree of glucose intolerance that either commences or is first diagnosed in pregnancy. The incidence of this condition is increasing, reflecting the increasing prevalence of obesity and metabolic syndrome. 

It causes maternal and fetal complications, including pre-eclampsia, polyhydramnios, fetal macrosomia, shoulder dystocia, birth trauma, neonatal metabolic complications, and perinatal death [2]. The pathogenetic mechanisms underlying GDM involve an imbalance between the capacity of the pancreatic β-cells and the increased demands for insulin due to decreased insulin sensitivity during pregnancy [3].

Polycystic ovary syndrome (PCOS) is the most common reproductive disorder affecting women of childbearing age [4], and it has been associated with an increased risk of GDM. According to the Rotterdam criteria, a diagnosis of PCOS requires the presence of at least two out of the following three features: (a) oligo or anovulation, (b) clinical and/or biochemical signs of hyperandrogenism, and (c) polycystic ovarian morphology on ultrasound, defined as ≥12 follicles measuring 2-9 mm in diameter and/or an ovarian volume >10 ml in at least one ovary. These features, particularly oligo/anovulation and hyperandrogenism, contribute to insulin resistance, a common pathway leading to GDM. The symptoms commonly associated with PCOS include amenorrhea, oligomenorrhea, hirsutism, obesity, infertility, anovulation, and acne. Obesity, also a risk factor for GDM, is prevalent in women with PCOS, compounding the risk. A frequent feature of PCOS, especially in obese women, is hyperinsulinemia, which also plays a central role in the development of GDM. Additionally, women with PCOS tend to exhibit elevated levels of plasminogen activator inhibitor-I, a factor implicated in early pregnancy loss [5], which may be exacerbated in pregnancies complicated by GDM. GDM itself is associated with a tenfold increased risk of progressing to type 2 diabetes mellitus. Moreover, maternal hyperglycemia during pregnancy may expose the offspring to a higher risk of autism spectrum disorders, obesity, hypertension (HTN), and cardiovascular disease [6].

PCOS has a global prevalence of approximately 1 in every 15 women and carries significant implications for pregnancy outcomes. These women are at increased risk of perinatal morbidity, including GDM, pregnancy-induced hypertension, and pre-eclampsia. Notably, PCOS increases the risk of developing GDM independently of the degree of adiposity [7]. This elevated risk is reflected in data indicating that approximately 40% of women with PCOS are at risk of developing GDM and other pregnancy-related complications [8]. In comparison, the global estimated burden of GDM in the general population is currently about 14% [9], highlighting the disproportionately higher risk among women with PCOS.

Maintaining adequate glycemic control during both the pre-pregnancy and pregnancy periods is essential for improving obstetric outcomes, as preexisting diabetes mellitus (DM) in pregnancy is associated with an increased risk of congenital malformations, stillbirth, and macrosomia [10]. There are shared etiopathological and metabolic features between PCOS and GDM, such as insulin resistance, dyslipidemia, and obesity, suggesting a common underlying mechanism [2,11]. Hypothyroidism also shares these metabolic disturbances, reinforcing the observed correlation between PCOS, thyroid dysfunction, and GDM incidence [11].

The idea behind this study is that early identification of women at higher risk (those with both PCOS and GDM) may allow for the implementation of targeted interventions before pregnancy or during early pregnancy. This proactive approach could help reduce the incidence of complications, both in the short term (during pregnancy) and the long term (postpartum). Additionally, identifying key risk factors will allow for the development of early interventions such as lifestyle modifications, medication, or closer monitoring of the pregnancy, which could reduce the risks associated with these conditions.

## Materials and methods

This prospective cohort study was conducted on 88 singleton pregnancies. The study was performed at AIIMS Raipur Obstetrics and Gynecology Department from January 2023 to December 2023 for 12 months.

Inclusion criteria

The inclusion criteria include: (i) Patients willing to participate in the study; (ii) Singleton pregnancy with a known case of GDM; (iii) GDM with diagnosed PCOS according to Rotterdam's criteria in the prenatal period; (iv) Maternal age >18 years; (v) Gestational age 5 weeks-40 weeks.

Exclusion criteria

The exclusion criteria include: (i) Patients not willing to participate in the study; (ii) Multiple pregnancies; (iii) History of maternal diseases like congenital adrenal hyperplasia, malignant ovarian tumors, Cushing’s syndrome, cardiac disease, renal disease, and hyperprolactinemia (patient will be diagnosed with the above-mentioned diseases with the help of history and clinical features); (iv) Insulin-dependent DM or diagnosed case of type 2 diabetes mellitus.

Brief methodology

After obtaining ethics committee clearance, the study was conducted on women visiting the outpatient department and admitted patients in the department who fulfilled the inclusion and exclusion criteria. Informed written consent was obtained from all the patients enrolled in the study. Patients enrolled in the study were divided into two groups: Group A: GDM with PCOS and Group B: GDM without PCOS. Detailed history of the participants (including chief complaints, history of present pregnancy, past medical/surgical /drug history, family history, menstrual history, obstetric history, immunization history, occupational history) along with demographic details was filled in the case record proforma (CRP). History suggestive of PCOS was asked, for example, irregular menstrual cycle, weight gain, acne, excessive hair growth, and ultrasound findings were noted to make the diagnosis of PCOS by Rotterdam's criteria. Rotterdam's criteria are defined by the presence of at least two of the following three features: (i) oligo or anovulation, (ii) clinical and/or biochemical signs of hyperandrogenism, and (iii) polycystic ovarian morphology on ultrasound, defined as having ≥12 follicles measuring 2-9 mm in diameter and/or an ovarian volume >10 ml in at least one ovary. Past history of infertility, ovulation induction, mode of conception, surgical history considering laparoscopic or diagnostic hysteroscopy for infertility, drug history including intake of metformin or insulin, and family history of diabetes were filled in the CRP.

At the first visit, we initially screened patients using the DIPSI criteria, which involves a non-fasting 75g glucose challenge test. Patients with a two-hour plasma glucose level of 140 mg/dL or above were identified as positive. Subsequently, these patients underwent a standard oral glucose tolerance test (OGTT) for further evaluation and confirmation of glucose intolerance. The diagnostic criteria for GDM on the basis of the OGTT include the cutoff of glucose values for fasting was ≥92 mg/dL, 1 h after fasting was ≥180 mg/dl, and 2 h after fasting ≥153 mg/dl. DIPSI includes non-fasting 75g glucose with a cut-off of ≥140mg/dl after two hours. A review of all the investigations, including previous ANC records, was done. Clinical examination was conducted, which included general physical examination, including height, weight and BMI; systemic examination and obstetric examination findings were documented in the CRP.

All the patients were advised to make routine ANC visits as per the institutional protocol. The diagnosed GDM patients were evaluated by an endocrinologist and the admission and drug therapy were decided according to the Institutional protocol. In every visit, the patient was checked for weight gain, blood pressure, fundal height on per abdominal examination, abdominal girth (cm), symphysio-fundal height (cm), and fetal heart rate. Patients were counselled to keep a strict check on the perception of fetal movement during every visit (daily fetal movement count charting was explained to the patient. A non-stress test was performed weekly/biweekly after 32 weeks of gestation. Fetal surveillance in the form of dating scan, aneuploidy screening, early and late anomaly scans, and growth scan with Doppler was studied. Pregnancy was monitored for the development of maternal and fetal complications like maternal development of hypertensive disorders in pregnancy, preterm delivery, polyhydramnios, oligohydramnios, fetal growth restriction or macrosomia, shoulder dystocia, and puerperal complications like postpartum hemorrhage, lactation failure, and breast abscess.

Women participating in the study were followed till delivery. At the time of delivery, gestational age, mode of delivery, mode of onset of labor-spontaneous/induced, caesarean section-emergency/elective, indication of caesarean section, preterm delivery, requirement of insulin or metformin or both, postpartum hemorrhage, postpartum wound infection, breast abscess, and lactation failure were studied.

Neonatal parameters like neonatal weight, Apgar score at 1 min and 5 min, meconium aspiration, respiratory distress syndrome, fetal acidosis, macrosomia, neonatal icterus, neonatal hypoglycemia, neonatal intensive care unit admission, and its indication were studied. Neonatal hyperviscosity was defined as a venous hematocrit (Hct) greater than 65%. Neonates were observed for the symptoms of neonatal hyperviscosity such as poor feeding, lethargy, respiratory distress, cyanosis, and hypoglycemia seizures (in severe cases).

Data was collected in the two subgroups and analyzed. The outcome of delivery and the neonatal outcomes were studied in the two groups, and the outcomes of the two groups (GDM with PCOS group and GDM without PCOS) were compared.

Sample size calculation

Patients were divided into two groups: Group A: GDM with PCOS group and Group B: GDM without PCOS group. Based on a previously published study (Foroozanfard et al. [2]), the sample size is estimated based on the outcome, the percentage of patients with pregnancy-induced hypertension is 27.7% in the GDM +PCOS group (Group A) and 13.7% in the GDM only group (Group B). The sample size was estimated using the following formula:

$N-[Z_(a/2) &radic;(2p(1-p)) +Z_(1-&beta;) &radic; (p_1 (1-p_1) p_2 (1-p_2))] ^2/〖(p_1-p_2) 〗^2$

Where p1 and p2 are the percentage of patients with pregnancy-induced hypertension in Group A (0.277) and Group B (0.137); p is the average of the proportions (0.277+0.137/2); Z α/2 =1.96 at 95% confidence interval; Z1-β=0.84 at 80% power. Substituting these values in the formula, the total sample size estimated is 88 in two groups, with 44 in each group.

Statistical analysis was carried out using IBM SPSS Statistics for Windows, Version 20 (Released 2011; IBM Corp., Armonk, New York, United States). Continuous and categorical variables were expressed as mean±SD and percentages, respectively. Categorical variables were compared using the chi-square test. Continuous variables were compared using Student's t-test. Two-sided P-values were considered statistically significant at P<0.05.

## Results

Table 1 compares maternal characteristics between women in Group A and Group B. Maternal age showed no significant difference between the groups (P=0.18, mean difference=1.47, 95% CI -0.69 to 3.64). Women with PCOS had significantly higher maternal weight in the first trimester (P=0.044, mean difference=4.12, 95% CI- 0.11 to 8.14) and borderline higher BMI (P=0.057, mean difference=1.25, 95% CI 0.03 to 2.54)

**Table 1:** Maternal Characteristics Table comparing maternal characteristics between women with gestational diabetes mellitus (GDM) with and without polycystic ovary syndrome (PCOS)

Maternal Characteristics	Group A (n=44)	Group B (n=44)	P-Values
Maternal age (years)	30.86±5.92	29.38±4.16	0.18
Maternal weight (kg) (mean ±SD)	65.85± 10.22	61.72± 8.65	0.044
Maternal BMI (mean ±SD)	26.01± 2.98	24.76± 3.11	0.057
Duration of marriage (years) (mean ±SD)	6.51 ±4.40	4.53 ±3.01	0.016
Weight gain in pregnancy (kg) (mean ±SD)	13.27±1.54	11.77±2.06	0.000
Gravidity N (%)			
Primigravida	18(40.9%)	19(44.2%)	0.82
Multigravida	26(59.1%)	25(56.8%)	
Gravidity			
G1	18(40.9%)	19(44.2%)	0.136
G2	11(25%)	16(37.2%)	
G3	9(20.5%)	8(18.6%)	
G4	5(11.4%)	0(0%)	
G5	0(0%)	0(0%)	
G6	1(2.3%)	0(0%)	

They also had a longer duration of marriage (P=0.016, mean difference=1.97, 95% CI 0.37 to 3.57) and a longer duration was required for conception. They gained more weight during pregnancy (P=0.000, mean difference=1.50, 95% CI 0.72 to 2.27). Gravidity did not differ significantly (P=0.82, OR=0.91, 95% CI 0.39 to 2.12).

The data in Table 2 examines comorbidities between the two groups, detailing the prevalence of various conditions such as DM, HTN, thyroid disorders, obesity, sickle cell trait (SCT), asthma, and associated conditions like acne. Comorbidities overall were not significantly significant (P=0.38, OR=0.68, 95% CI 0.29 to 1.61), an associated condition like acne was statistically significant in the PCOS group (P=0.02).

**Table 2:** Distribution Based on Comorbidities and Family History Table examining comorbidities between two groups, detailing the prevalence of various conditions such as diabetes mellitus (DM), hypertension (HTN), thyroid disorders, obesity, sickle cell trait (SCT), asthma, and acne. ^#^Pearson’s chi-square test.

Comorbidities, N(%)	Group A (n=44)		Group B (n=44)	P-Values
No	24(54.5%)		28(63.6%)	0.38^#^
Yes	20(45.5%)		16(36.4%)	
Comorbidities, N(%)				
DM	3(6.8%)		0(0%)	0.07^#^
HTN	1(2.3%)		0(0%)	0.31^#^
Thyroid (hypothyroidism)	13(29.5%)		9(20.4%)	0.32^#^
Obesity	4(9.1%)		4(9.1%)	1^#^
SCT	0(0%)		2(4.5%)	0.17^#^
Asthma	0(0%)		1(2.3%)	0.31^#^
Acne	5(11.3%)		0(0%)	0.02^#^
Family H/O N(%)				
DM	12(27.2%)		5(11.3%)	0.05^#^
HTN	1(2.3%)		2(4.5%)	0.55^#^
DM HTN	6(13.6%)		5(11.3%)	0.74^#^
Thyroid (hypothyroidism)	1(2.3%)		0(0%)	0.31^#^
Family h/o of cardiovascular disease, metabolic disorders like dyslipidemia N(%)				
Yes	20(45.5%)		12(27.2%)	0.07#
No	24(54.5%)		32(72.7%)	

The data compares family histories of DM, HTN, both conditions, and thyroid disorders between the two groups. Notably, 12 participants (27.2%) in one group had a family history of DM, compared to 5 (11.3%) in the other, showing a borderline significant difference with a p-value of 0.05. In contrast, the prevalence of HTN was similar between the groups, with p-values indicating no significant differences for HTN (p=0.55), both DM and HTN (p=0.74), and thyroid disorders (P=0.31). The findings suggest the family history of DM also was not statistically significant (P=0.05, OR=2.22, 95% CI 0.91 to 5.41).

Women with PCOS were significantly more likely to require higher doses of insulin (P=0.001, OR=4.66, 95% CI 1.81 to 11.97). No significant differences were found in fasting blood glucose levels (P=0.55), 1-hour OGTT (P=0.66), 2-hour OGTT (P=0.80), or HbA1c at diagnosis (P=0.76).

Table 3 shows that the fasting blood glucose on OGTT in Group A was 93.6 mmol/L as compared to Group B with the value 95.61 mmol/L (T=-0.59, P=0.55, mean diff=-1.97, 95% CI (-8.53-4.58). The 1-hour and 2-hour blood glucose levels were also less in Group A as compared to Group B (P=0.66, mean diff=-4.02, 95% CI (-22.69-14.64). HbA1C is slightly higher in GDM with PCOS group 5.85 as compared to GDM without PCOS group 5.79 (T=0.30, P=0.76, mean diff=0.06, 95% CI (-0.37-0.50). Twenty-one patients did not require any medications in Group B, as compared to Group A, where 12 patients had no requirement for medications. A combination of insulin and metformin was required in 3 out of 44 patients in the GDM with PCOS group, as compared to GDM without PCOS patients where only one patient required a combination therapy of Insulin and Metformin. In Group A patients, 24 out of 44 required an increase in insulin dosage, compared to only nine patients in Group B who needed a dose adjustment.

**Table 3:** Distribution Based on Mean Sugar Values and HbA1c at Diagnosis and Management During Pregnancy Table providing information about mean sugar values and HbA1c at diagnosis and management during pregnancy ^#^Pearson's chi-square test OGTT: Oral glucose tolerance test

Mean Blood Glucose and HbA1c Values	Group A (n=44)	Group B (n=44)	P-Values
Fasting blood glucose on OGTT (mmol/L) (mean ±SD)	93.6 ±12.24	95.61±18.14	0.55
1-hour blood glucose on OGTT (mmol/L) (mean ±SD)	166.9 ±40.61	171 ±47.20	0.66
2-hour blood glucose on OGTT (mmol/L) (mean ±SD)	147.1 ±34.57	149.3± 47.6	0.80
HbA1c at diagnosis (mean ±SD)	5.85±1.01	5.79±1.07	0.76
Management during current pregnancy N (%)			
Nil medications	12(27.2%)	21(47.7%)	0.16^#^
Insulin only	25(56.8%)	17(38.6%)	
Metformin only	4(9.1%)	5(11.3%)	
Combination	3(6.8%)	1(2.2%)	
Insulin dose increased N (%)			
Yes	24(54.5%)	9(20.4%)	0.001^#^
No	20(45.5%)	35(79.5%)	

In the management of GDM, metformin was typically initiated at a dose of 500 mg once or twice daily and gradually titrated based on glycemic control and patient tolerance, with an average effective dose ranging from 1500 to 2000 mg per day.

In our study population, the average insulin dose in the insulin monotherapy group ranged from 20 to 40 units per day, while those in the combined therapy group (metformin plus insulin) required a lower dose, typically between 10 and 30 units per day, depending on individual glycemic control needs.

Table 4 shows the GDM with PCOS group has undergone caesarean section in 36 patients, as compared to the GDM without PCOS with 27 caesarean sections (P=0.04). Elective caesarean section was seen in 21 patients in the GDM with PCOS group (Group A) as compared to the GDM without PCOS group (Group B), with 12 elective caesarean sections (P=0.27).

**Table 4:** Distribution Based on the Mode of Delivery Table demonstrating the mode of delivery in both the groups *Pearson’s chi-square test ^#^Fisher's test LSCS: Lower segment caesarean section; GDM: gestational diabetes mellitus; PCOS: polycystic ovary syndrome

Mode of Delivery N(%)	GDM with PCOS (n=44)	GDM without PCOS (n=44)	Test values
Vaginal	8(18.2%)	16(36.36%)	X^2^=3.94, P=0.04*
LSCS	36(81.8%)	27(61.36%)	
Vaginal delivery N (%)			
Spontaneous	4(9.09%)	9(20.45%)	X^2^=0.08, P=0.77^#^
Induction of labor	4(9.09%)	7(15.9%)	
LSCS N (%)			
Emergency	15(34.09%)	15(34.09%)	X^2^=1.19, P=0.27*
Elective	21(47.72%)	12(27.27%)	
No	42(95.4%)	44(100%)	

Table 5 compares the preeclampsia and neonatal outcomes between women with Group A and those with Group B, each group comprising 44 participants.

**Table 5:** Association with Pre-eclampsia and Neonatal Outcomes *Pearson’s chi-square test ^#^Fisher's test NICU: Neonatal intensive care unit; RDS: respiratory distress syndrome; IUD: intrauterine death; MAS: meconium aspiration syndrome; IVH: intraventricular hemorrhage

Pre-eclampsia N (%)	Group A (n=44)	Group B (n=44)	P -values
Yes	7(15.9%)	3(6.8%)	X^2^=1.80, P=0.17 OR=2.58 95% CI (0.62-10.7)^#^
No	37(84.09%)	41(93.1%)	
Neonatal outcomes	GROUP A (n=44)	GROUP B (n=44)	Test values
Birth weight (kg)			
Macrosomia N (%)			X^2^=0.34, P=0.55^#^
Yes	2(4.5%)	1(2.3%)	
No	42(95.5%)	43(97.7%)	
APGAR <7 at 1min N (%)	3(6.8%)	5(11.3%)	X^2^=0.55, P=0.45^#^
APGAR <7 at 5 min N (%)	0	0	-
NICU admission			
Yes	5(11.3%)	4(9.1%)	X^2^=0.12, P=0.72^#^
No	39(88.6%)	40(90.9%)	
RDS			
Yes	1(2.2%)	0(0%)	X^2^=1.01, P=0.31^#^
No	43(97.7%)	44(100%)	
Hypoxia & acidosis			
Yes	1(2.2%)	1(2.2%)	X^2^=0, P=1^#^
No	43(97.7%)	43(97.7%)	
IUD			
Yes	1(2.2%)	0(0%)	X^2^=1.01, P=0.31^#^
No	43(97.7%)	44(100%)	
Still birth			
Yes	1(2.2%)	0(0%)	X^2^=1.01, P=0.31^#^
No	43(97.7%)	44(100%)	
MAS			
Yes	1(2.2%)	0(0%)	X^2^=1.01, P=0.31^#^
No	43(97.7%)	44(100%)	
Neonatal hypoglycemia			
Yes	1(2.2%)	0(0%)	X^2^=1.01, P=0.31^#^
No	43(97.7%)	44(100%)	
Neonatal hypocalcemia			
Yes	0(0%)	0(0%)	-
No	44(100%)	44(100%)	
Neonatal jaundice			
Yes	4(9.1%)	1(2.2%)	X^2^=1.91, P=0.16^#^
No	40(90.9%)	43(97.7%)	
Sepsis			
Yes	1(2.2%)	0(0%)	X^2^=1.01, P=0.31^#^
No	43(97.7%)	44(100%)	
Hyper viscosity syndrome			
Yes	1(2.2%)	0(0%)	X^2^=1.01, P=0.31^#^
No	43(97.7%)	44(100%)	
IVH			
Yes	2(4.5%)	1(2.2%)	X^2^=34, P=0.55^#^
No	42(95.4%)	43(97.7)	
Yes	2(4.5%)	0(0%)	X^2^=2.04, P=0.15^#^

The table shows that 15.9% (7 out of 44) of individuals with Group A had pre-eclampsia, compared to 3(7%) (X² = 1.80, P = 0.17) of those with Group B.

Neonatal outcomes were generally similar across both groups, but a few notable observations were made. The incidence of macrosomia was reported in two patients (4.5%) in the GDM with PCOS group and one patient (2.3%) in the GDM without PCOS group, with no significant difference between the two (p=0.55). Additionally, APGAR scores at both one and five minutes did not show significant discrepancies, as three patients (6.8%) in the PCOS group and 5 (11.3%) in the non-PCOS group scored less than 7 at one minute (P=0.45), indicating that overall neonatal health was comparable between the two groups.

NICU admissions were also similar, with 5 (11.3%) of the GDM with PCOS group (Group A) requiring neonatal care, compared to 4 (9.1%) in the non-PCOS group (P=0.72). Other complications, such as respiratory distress syndrome (RDS), hypoxia and acidosis, intrauterine death (IUD), stillbirth, and neonatal hypoglycemia, were reported with low frequencies and did not show significant association. One participant (2.2%) in the GDM with PCOS group experienced RDS, while none were reported in the non-PCOS group (p=0.31) which indicate nonsignificant association.

However, neonatal jaundice was more frequently observed in the GDM with PCOS group, affecting four participants (9.1%) compared to one participant (2.2%) in the non-PCOS group, though this difference was not statistically significant (P=0.16). Congenital anomalies were reported in two participants (4.5%) in the GDM with PCOS group, whereas none were noted in the non-PCOS group (P=0.15) but the results did not denote statistical significance.

## Discussion

PCOS is an endocrine disorder affecting women of childbearing age, with an incidence of 5 to 10 percent. The key pathophysiological features include hyperandrogenism, insulin resistance, and persistent anovulation. Clinically, it manifests as obesity, hirsutism, infertility, and menstrual irregularities. The incidence of GDM is also increasing in our country. Both PCOS and GDM are associated with adverse maternal and perinatal outcomes.

We observed that women in Group A had a higher maternal weight gain compared to those in Group B. BMI, HbA1C, and total cholesterol are known predictors of developing GDM in patients with PCOS [6]. The insulin resistance commonly found in PCOS contributes to increased conversion of glucose to fat, which can further exacerbate weight gain.

In our study, the mean BMI in Group A was 26.01, compared to 24 in Group B. However, this difference was not statistically significant. Nonetheless, the study did reveal a statistically significant difference in maternal weight gain between the GDM with PCOS group and the GDM-only group.

The increased risk of GDM in women with PCOS has been linked to both obesity and advanced maternal age (Mustaniemi et al. [7]). Supporting this, previous studies have observed higher BMI and statistically significant weight gain in women with both PCOS and GDM (Manoharan et al. [12]). PCOS is associated with various comorbidities that contribute to obesity and long-term metabolic consequences. Among these, insulin resistance plays a central role, predisposing individuals with PCOS to develop GDM [7,12]. There is a strong correlation between having a family history of diabetes and the development of GDM, particularly in patients with PCOS. The family history of Diabetes Mellitus in first-degree relatives was associated with a higher incidence of GDM in the GDM with PCOS group as compared to the GDM-only group. In the study by Ouyang et al. [6], logistic regression revealed that family history was an independent risk factor for the development of GDM in patients with PCOS. Similarly, a systematic review conducted by Slouha et al. [13] confirmed the development of GDM in PCOS patients with a family history of diabetes.

In addition to family history, comorbid hypothyroidism also showed an association with the development of GDM in PCOS patients. A study by Ashrafi et al. [14] found a similar association between the development of GDM in the PCOS group and the comorbidity of hypothyroidism. However, very few studies have indicated hypothyroidism as a strong predictor for developing GDM in PCOS patients.

This study reveals that patients in both the GDM with PCOS group and the GDM-only group did not show a significant difference in fasting, postprandial, and HbA1C levels. However, the requirement for combined therapy (metformin plus insulin) was higher in the GDM with PCOS group. Additionally, the dose of insulin increased during the course of pregnancy in the GDM with PCOS group.

In terms of glucose levels, Mahoharan et al. [12] found higher mean fasting glucose levels, unlike the present study, but there was no significant impact on postprandial glucose levels and HbA1C, which is similar to our findings. Slouha et al. [13] also observed increased fasting blood glucose, fasting insulin, and HbA1C levels, which contrasts with the findings of our study. Ouyang et al. [6] reported higher HbA1C values and identified it as an independent risk factor for GDM development in PCOS patients.

In our study, there was a higher incidence of preeclampsia in the GDM with PCOS group; however, it was not statistically significant. In the study by Fougner et al. [15], women with GDM did not show an association with any maternal complications, such as the development of preeclampsia or postpartum hemorrhage. Some studies have identified PCOS as a contributing factor to an increased risk of developing complications like preeclampsia [12]. The systematic review also demonstrated that women with both GDM and PCOS were more likely to develop pregnancy-induced hypertension compared to those in the GDM-only group [13]. Other studies have also linked PCOS to a likelihood of preeclampsia during pregnancy [3,4].

Neonates born to mothers in the GDM with PCOS group were more likely to have low birth weight. However, in contrast to our findings, the study conducted by Valdimarsdottir et al. [9] reported a higher rate of stillbirths in women with PCOS, whereas our study did not find any statistically significant association between PCOS and stillbirth.

Both PCOS and GDM have been linked to an increased risk of neonatal complications such as hypoglycemia and low APGAR scores [2]. Nevertheless, no strong correlation has been established between the coexistence of GDM with PCOS and other neonatal complications. In our study, we observed a slightly higher incidence of low APGAR scores (<7) in the GDM-only group (5%) compared to the GDM with PCOS group (3%). Additionally, the incidence of macrosomia was marginally higher in the GDM with PCOS group (2%) than in the GDM-only group (1%). However, these differences were not statistically significant.

Importantly, there were no cases of neonatal hypoglycemia reported in either group. Furthermore, our study found no association between GDM with PCOS and other neonatal complications, including hypoxia, acidosis, neonatal jaundice, sepsis, hyperviscosity syndrome, and congenital anomalies.

## Conclusions

This study found no significant difference in fetomaternal outcomes between GDM patients with and without PCOS. Glycemic control, as reflected in fasting, postprandial, and HbA1C levels, was similar across groups. However, hypothyroidism was more prevalent in the GDM with PCOS group, suggesting a need for closer thyroid monitoring. A family history of diabetes was a strong predictor of GDM development in women with pre-existing PCOS. Additionally, these patients more frequently required combined therapy with insulin and metformin, with higher insulin doses needed as pregnancy progressed. These findings highlight the importance of personalized care in managing GDM among women with PCOS.

## References

[1] Joham AE, Ranasinha S, Zoungas S, Moran L, Teede HJ (2014). Gestational diabetes and type 2 diabetes in reproductive-aged women with polycystic ovary syndrome. J Clin Endocrinol Metab.

[2] Foroozanfard F, Moosavi SG, Mansouri F, Bazarganipour F (2014). Obstetric and neonatal outcome in PCOS with gestational diabetes mellitus. J Family Reprod Health.

[3] Holte J, Gennarelli G, Wide L, Lithell H, Berne C (1998). High prevalence of polycystic ovaries and associated clinical, endocrine, and metabolic features in women with previous gestational diabetes mellitus. J Clin Endocrinol Metab.

[4] Li G, Huang W, Zhang L (2018). A prospective cohort study of early-pregnancy risk factors for gestational diabetes in polycystic ovarian syndrome. Diabetes Metab Res Rev.

[5] Homburg R (2006). Pregnancy complications in PCOS. Best Pract Res Clin Endocrinol Metab.

[6] Ouyang P, Duan S, You Y, Jia X, Yang L (2023). Risk prediction of gestational diabetes mellitus in women with polycystic ovary syndrome based on a nomogram model. BMC Pregnancy Childbirth.

[7] Mustaniemi S, Vääräsmäki M, Eriksson JG (2018). Polycystic ovary syndrome and risk factors for gestational diabetes. Endocr Connect.

[8] Choudhury AA, Rajeswari VD (2022). Polycystic ovary syndrome (PCOS) increases the risk of subsequent gestational diabetes mellitus (GDM): a novel therapeutic perspective. Life Sci.

[9] Valdimarsdottir R, Vanky E, Elenis E (2024). Polycystic ovary syndrome and gestational diabetes mellitus association to pregnancy outcomes: a national register-based cohort study. Acta Obstet Gynecol Scand.

[10] Hincapie MA, Badeghiesh A, Baghlaf H, Dahan MH (2025). Association between pre-gestational diabetes in women with polycystic ovary syndrome and adverse obstetric outcomes. Eur J Obstet Gynecol Reprod Biol.

[11] Quaresima P, Myers SH, Pintaudi B, D'Anna R, Morelli M, Unfer V (2025). Gestational diabetes mellitus and polycystic ovary syndrome, a position statement from EGOI-PCOS. Front Endocrinol (Lausanne).

[12] Manoharan V, Wong VW (2020). Impact of comorbid polycystic ovarian syndrome and gestational diabetes mellitus on pregnancy outcomes: a retrospective cohort study. BMC Pregnancy Childbirth.

[13] Slouha E, Alvarez VC, Gates KM, Ankrah NM, Clunes LA, Kollias TF (2023). Gestational diabetes mellitus in the setting of polycystic ovarian syndrome: a systematic review. Cureus.

[14] Ashrafi M, Sheikhan F, Arabipoor A, Hosseini R, Nourbakhsh F, Zolfaghari Z (2014). Gestational diabetes mellitus risk factors in women with polycystic ovary syndrome (PCOS). Eur J Obstet Gynecol Reprod Biol.

[15] Fougner SL, Vanky E, Løvvik TS, Carlsen SM (2021). No impact of gestational diabetes mellitus on pregnancy complications in women with PCOS, regardless of GDM criteria used. PLoS One.

